# Mapping the landscape of small-scale and backyard poultry flocks in the Netherlands: a nationwide survey for One Health surveillance

**DOI:** 10.1016/j.onehlt.2026.101626

**Published:** 2026-10-23

**Authors:** J.J. Oosters, M. van Boven, M.J.M. Bonten, J.A. Stegeman, F.C. Velkers

**Affiliations:** a Julius Center for Health Sciences and Primary Care, Department of Epidemiology, University Medical Center Utrecht, Str 6.131, P.O. Box 85500, 3508 GA, Utrecht, the Netherlands; b Department of Population Health Sciences, Faculty of Veterinary Medicine, Utrecht University, Yalelaan 7, 3584 CL Utrecht, the Netherlands

**Keywords:** Influenza A virus, Backyard poultry, Zoonoses, One Health, Epidemiology

## Abstract

Small-scale and backyard poultry systems are increasingly common in high-income countries but remain poorly characterised in animal health surveillance and zoonotic risk assessments. Operating at the wildlife-domestic animal-human interface, these systems may contribute to the transmission of zoonotic pathogens such as avian influenza viruses (AIVs). This study provides the first nationwide characterisation of small-scale and backyard poultry systems in the Netherlands using survey data from more than 3500 respondents. The survey revealed substantial heterogeneity among small-scale poultry keepers, and identified four distinct groups, differing in demographics, level of organisation, flock size and composition, housing practices, and motivations. Backyard flocks formed the largest group, typically consisting of small chicken flocks of up to ten birds (83.8%) for hobby or egg production, whereas other holding types kept larger flocks with more diverse and function-specific motivations. Chickens were the most commonly kept species, however, mixed-species flocks, including combinations of Galliformes and Anseriformes, were also frequently reported, particularly by petting and care farms (60%). Poultry were most often housed using a combination of indoor night shelters and outdoor access, with a substantial proportion of respondents reporting free-range systems (41%), which may increase exposure to wild birds that act as AIV reservoirs. Respondents were widely distributed across the Netherlands, with higher representation in rural and suburban areas, though holdings were also present in urban areas. These findings highlight the importance of including small-scale and backyard poultry systems in surveillance and risk assessment frameworks to improve zoonotic disease prevention and control strategies.

## Highlights

• Backyard poultry flocks are poorly characterised in zoonotic risk assessments.
• First nationwide characterisation of Dutch small-scale poultry systems.
• Four holding types differ in flock size, species mix, housing, and motives.
• Chickens are the most kept species, mixed-species flocks were also common.
• Free-range management was common among backyard poultry keepers.

## 1 Introduction

Over the past decades, small-scale and backyard poultry-keeping has increased in several high-income countries, including France, the United Kingdom, and the United States. This trend has been attributed to increasing interest in improved food quality, sustainability, animal welfare, and keeping chickens as companion animals [1], [2], [3]. Next to private backyard flocks, poultry is also kept in small-scale systems. These include settings such as exhibition breeding or the small-scale production of poultry products. In addition, poultry is also kept in locations such as petting zoos or care farms. More broadly, poultry-keeping has diversified, including initiatives such as community-supported agriculture [4], mobile poultry housing [5], [6], and other short food supply chains in which products are sold directly to consumers or through a single intermediary [7]. As a result, poultry-keeping increasingly serves a wide range of functions, including small-scale food production, recreational, educational, and companion animal roles [8].

In several European countries, including Germany, France, the United Kingdom, and Ireland, anyone who keeps poultry, including small-scale and backyard flock owners with only one bird, is required to register their flocks [9], [10], [11], [12]. In the Netherlands, poultry owners are only required to register their flock if they exceed 250 birds. Registration is also required for smaller flocks if poultry products (e.g., eggs) are sold, to ensure traceability for food safety purposes [13]. Flocks smaller than 250 birds without commercial activity are considered backyard flocks and are not obliged to report animal movements [14]. According to the World Organisation for Animal Health (WOAH), there is no globally standardised definition of backyard poultry, which complicates the inclusion of these holdings in policy frameworks and surveillance systems [15]. Consequently, small-scale and backyard poultry holdings remain poorly documented, and structured data on this population are limited [2], [3], [16], [17].

While animal health monitoring and surveillance in high-income countries primarily focus on commercial farms, small-scale and backyard flocks remain poorly characterised. This gap represents a potential blind spot in current surveillance systems, particularly as these flocks may be exposed to infectious agents and zoonotic pathogens. Strengthening our understanding of this sector is therefore urgent for improving animal health monitoring and preparedness [8], [16].

Animal health monitoring and surveillance are especially important for pathogens affecting both poultry and human health. These include endemic zoonotic pathogens, such as *Salmonella* and *Campylobacter*, as well as emerging or re-emerging threats such as avian influenza viruses (AIVs) and Newcastle disease virus (NDV) [18], [19], [20]. For example, *Campylobacter jejuni* was detected in 45% of Swedish backyard holdings [21]. Campylobacteriosis and salmonellosis remain among the most reported zoonoses in the European Union [18]. Close human-bird contact in small-scale systems, combined with limited biosecurity practices, may increase opportunities for pathogen exposure through handling birds, eggs, litter/manure, and dust, particularly via faecal-oral or respiratory transmission routes [22], [23].

Small-scale and backyard animal holdings operate at the interface between domestic animals, humans, and the environment, making them potentially relevant for pathogen transmission across species and ecological domains. A study from Finland reported low numbers of AIV seropositive backyard flocks [24]. In contrast, a study in France reported a seroprevalence of 25.7% for AIV and 11.4% for H5-specific AIV in backyard flocks [25]. In the Netherlands, between October 2021 and January 2023, 91 poultry-locations tested positive for AIV, including both commercial poultry farm locations and other captive birds [26]. However, limited documentation of the small-scale and backyard flock sector in the Netherlands means that the proportion of infected holdings cannot be determined, making it difficult to assess the risks to the human population. Furthermore, pathogens, especially AIVs, evolve and new variants may emerge [27]. For example, highly pathogenic AIV H5N1 clade 2.3.4.4b has caused widespread outbreaks in wild birds and poultry globally and has recently shown spillover events into dairy cattle in the United States in 2024–2025 and in the Netherlands in 2026 [28], [29].

The Netherlands is known for its high human population density and has one of the highest densities of commercial poultry in Europe, making it a particularly relevant setting for One Health research. In addition, the country hosts large numbers of migratory waterbirds along the East Atlantic Flyway, further increasing the relevance of studying disease risks at the wildlife-domestic animal-human interface. Several studies have aimed to address critical knowledge gaps concerning small-scale and backyard flocks in their country, and the potential role of these flocks in infectious disease dynamics [1], [2], [3], [8], [16], [17]. The number of backyard poultry owners in the Netherlands was estimated to exceed 100,000 in 2004, collectively keeping more than 1,000,000 birds [30], in a country with a population size of around 16 million people at that time [31]. However, systematic data on these holdings in the Netherlands remain scarce.

Understanding the characteristics and spatial distribution of small-scale and backyard holdings is essential for assessing potential disease risks and improving surveillance strategies for both existing and emerging zoonoses. Describing poultry owners' characteristics, motivations, flock size and composition, and poultry housing can help identify how these holdings operate at the wildlife-domestic animal-human interface. This information contributes to building a clearer “public health map” of a previously hidden landscape: small-scale and backyard poultry in the Netherlands.

## 2 Materials and methods

### 2.1 Study design and survey development

We carried out a cross-sectional study to obtain a better understanding of the characteristics of Dutch small-scale and backyard poultry holdings. Data were collected through a nationwide, online questionnaire, conducted between March and September 2025.

The questionnaire was developed and distributed using Qualtrics (Provo, UT) [32]. Questions were informed by previous studies on backyard poultry holdings, covering flock characteristics, owner demographics, housing, as well as wild bird-poultry contact, biosecurity practices, and the owners' views on avian influenza mitigation measures [1], [2], [33], [34], [35].

The questionnaire consisted of 57 questions divided into six separate themes: 1) Poultry owner characteristics; 2) Flock characteristics; 3) Housing and husbandry practices; 4) Poultry-wildlife interactions; 5) Poultry-human contact; and 6) Avian influenza and mitigation measures. The full questionnaire is available in Supplementary File A. Individual variables for themes 1–3 are described in the subsection *Variables* and Supplementary File B (‘Variable descriptions’). Theme 4 was included because wild birds, especially wild waterfowl (Anseriformes), serve as the main reservoir for AIVs [27], [28]. Theme 5 covered factors that influence zoonotic transmission risk and theme 6 was included because the use of and owners' view on AIV mitigation measures are key determinants for the effectiveness of control measures in small-scale and backyard settings.

### 2.2 Target population and participant recruitment

The target population included small-scale and backyard poultry flock owners in the Netherlands. We included poultry owners who keep fowl species (Galliformes), waterfowl (Anseriformes), raptors, and/or ratites (Ratitae), considering the susceptibility of these species to AIV infection [36], [37], [38]. Survey respondents who, for example, kept only pigeons, were excluded based on the low susceptibility to AIV infection reported for pigeons [39]. The eligibility criteria for participants were as follows: participants had to keep at least one bird, be 18 years or older at the time of completing the questionnaire, reside and keep their flock in the Netherlands, and keep up to 250 birds.

The 250-bird cut-off was based on the current poultry registration legislation in the Netherlands, which requires poultry owners to register their flocks if they keep more than 250 birds [14]. A small number of participants who reported flock sizes larger than 250 birds were retained in the dataset, of whom two reported to keep fewer than 270 birds, and one holding kept 750 birds. These holdings were included in the data analyses because their responses to the survey questions did not appear to differ substantially from those of other comparable participants. The respondent keeping 750 birds self-identified as an ornamental fowl breeder and, apart from flock size, was not an obvious outlier within this group.

Upon opening the web link or scanning the QR-code directing participants to the online questionnaire, participants first received information about the study and how their data would be collected and used prior to the start of the survey. Before being able to access the survey questions, participants needed to tick a box, thereby confirming that they had read the information provided about the survey and consented to participation and to the use of their data for this study.

Participants were recruited using non-probability sampling methods [40], [41], including self-selection (volunteer sampling) and snowball sampling. The online questionnaire was disseminated via the research website of the Faculty of Veterinary Medicine of Utrecht University, the websites of various associations of backyard and small-scale animal keepers, social media platforms, as well as various newsletters. To encourage participation, survey respondents could win a €50 gift card. Details on participant recruitment channels are described in Supplementary File B (‘Participant recruitment channels’).

There is no official registry of small-scale and backyard flock owners in the Netherlands, but estimates suggest that total numbers may exceed 100,000 [30]. If true, a minimum sample of approximately 400 respondents would be required to estimate population proportions with a 5% margin of error under random sampling assumptions. Our study population exceeded this number by an order of magnitude. In section 2.4, we evaluate spatial representativeness using areal smoothing (BYM2-model) and comparison with self-reported estimates of local flock densities.

To reduce measurement and response bias, the questionnaire underwent several testing rounds by a panel consisting of veterinarians working in the field of small-scale holdings, poultry specialists, and social scientists. Panel members tested the questionnaire for clarity, effectiveness, question relevance, and whether questions were unbiased. Based on their feedback, questions were refined before dissemination. For multiple-choice questions, participants were given the option to select “other”, allowing them to specify and formulate their own response. Lastly, survey respondents were anonymous, and no responses could be traced back to specific individuals.

### 2.3 Variables

The key variables studied included *holding type*, *flock size*, *urbanisation level*, poultry keeping *motivation*, taxonomic *bird groups*, *mixed housing,* and *indoor-* and *outdoor housing*. Poultry owner characteristics were described using various variables, including *age, gender, education level, urbanisation level* of the postal code where the poultry holding was located (Table 1, Supplementary File B)*,* whether poultry owners were part of an *animal organisation* (e.g., breeder associations, hobbyist organisations, or petting farm networks), whether they kept poultry near their home or elsewhere (*poultry at home*), and the number of other pet species kept besides birds (*number other pet species*). The variables and their relevance are described in Supplementary File B (‘Variable descriptions’).

**Table 1:** Characteristics of poultry owners by self-identified holding types and comparison with the Dutch general population.

	Backyard (*n* = 3336)	Petting & care farm (*n* = 110)	Breeder (*n* = 42)	Small community farm (*n* = 37)	Total (*n* = 3525)	General population^4^
**Age**						
<20	5 (0.1%)	2 (1.8%)	1 (2.4%)	–	8 (0.2%)	–
20–39	348 (10.4%)	31 (28.2%)	9 (21.4%)	6 (16.2%)	394 (11.2%)	33.1%
40–64	1768 (53.0%)	65 (59.1%)	17 (40.5%)	15 (40.5%)	1865 (52.9%)	40.6%
65–79	1112 (33.3%)	9 (8.2%)	13 (31.0%)	7 (18.9%)	1141 (32.4%)	19.7%
≥80	99 (3.0%)	2 (1.8%)	2 (4.8%)	7 (18.9%)	110 (3.1%)	6.5%
Median [Min, Max]	60 [18, 97]	51 [18, 84]	61 [19, 82]	51 [33, 87]	60 [18, 97]	52.5 [0, ≥105]
**Gender**						
Female	1879 (56.3%)	73 (66.4%)	8 (19.0%)	7 (18.9%)	1967 (55.8%)	50.2%
Male	1429 (42.8%)	34 (30.9%)	34 (81.0%)	28 (75.7%)	1525 (43.3%)	49.8%
Prefer not to say	23 (0.7%)	2 (1.8%)	–	1 (2.7%)	26 (0.7%)	–
Missing	5 (0.1%)	1 (0.9%)	–	1 (2.7%)	7 (0.2%)	–
**Education**^1^						
Higher	1763 (52.8%)	50 (45.5%)	22 (52.4%)	22 (59.5%)	1857 (52.7%)	33.3%
Intermediate	1057 (31.7%)	54 (49.1%)	11 (26.2%)	10 (27.0%)	1132 (32.1%)	40.8%
Primary	498 (14.9%)	6 (5.5%)	9 (21.4%)	4 (10.8%)	517 (14.7%)	26.0%
Missing	18 (0.5%)	–	–	1 (2.7%)	19 (0.5%)	–
**Urbanisation level**^2^						
Urban	400 (12.0%)	60 (54.5%)	3 (7.1%)	7 (18.9%)	470 (13.3%)	56.7%
Suburban	1249 (37.4%)	34 (30.9%)	21 (50.0%)	15 (40.5%)	1319 (37.4%)	34.9%
Rural	1591 (47.7%)	15 (13.6%)	16 (38.1%)	15 (40.5%)	1637 (46.4%)	9.0%
Missing	96 (2.9%)	1 (0.9%)	2 (4.8%)	–	99 (2.8%)	0.6%
**Affiliated with association**^3^						
No	3156 (94.6%)	25 (22.7%)	5 (11.9%)	15 (40.5%)	3201 (90.8%)	–
Yes	180 (5.4%)	85 (77.3%)	37 (88.1%)	22 (59.5%)	324 (9.2%)	–
**Poultry at home**						
No	123 (3.7%)	76 (69.1%)	4 (9.5%)	16 (43.2%)	219 (6.2%)	–
Yes	3213 (96.3%)	34 (30.9%)	38 (90.5%)	21 (56.8%)	3306 (93.8%)	–
**Number other pet species**						
Median [Min, Max]	1.00 [0, 8.00]	5.00 [0, 11.0]	1.00 [0, 5.00]	1.00 [0, 5.00]	1.00 [0, 11.0]	–

1 Higher = higher education (professional or academic); Intermediate = upper secondary / intermediate occupational training; Primary = primary / lower secondary / entry-level occupational training.

2 Urban = surrounding address density above 1500 addresses per km^2^; Suburban = surrounding address density between 500 and 1500 addresses; Rural = surrounding address density below 500 addresses.

3 Affiliated with an animal organisation, e.g., *KLN* or *vSKBN (Supplementary File B)*.

4 General population data was derived from CBS and PC4 data [42], [46], [47], [48].

### 2.4 Statistics: spatial data analysis

The spatial distribution of survey respondents was derived from their four-digit postal codes, which were available for almost all respondents (*n* = 3446). The geospatial four-digit postal code information of the Netherlands was extracted from the publicly available PDOK CBS Postcode4 (2023) dataset (henceforth called PC4 data) [42]. The polygon centroids of the four-digit postal codes in the Netherlands were used to assign specific location data points to respondents. In the location maps (Fig. 1A and B), jitter (750 m) was applied to prevent data points from overlapping if originating from the same postal code polygon centroid. The same PC4 data [42] contained the urbanisation levels and surrounding address densities per km^2^ for each postal code region [43], which were used to create the three urbanisation levels (rural, suburban, and urban), as described in Supplementary File B (‘Variable descriptions’).

**Fig. 1:**
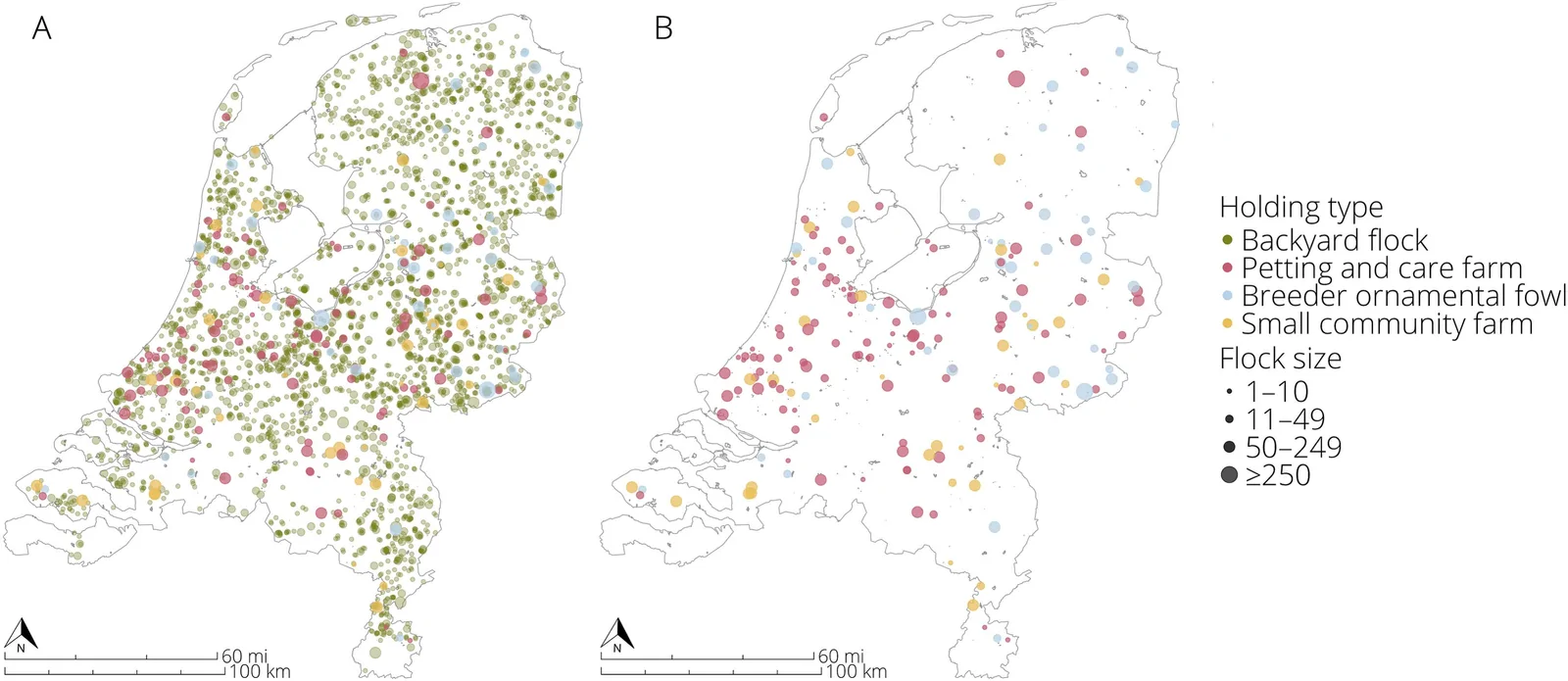
Geographical distribution of survey respondents stratified by self-identified holding type and flock size. A) Spatial distribution of all respondents, including backyard flock owners. B) Spatial distribution of respondents excluding backyard flock owners.

To model spatial variation in respondent intensity across the country, and to identify spatial clustering and potential undersampling, we used a BYM2-model (Besag-York-Mollié), of which the results are shown in Supplementary File C (Supplementary Fig. C.1A). BYM2 is a Bayesian spatial smoothing model for areal data, estimating smoothed relative risks [44]. In our analyses, relative risks represented an expected number of respondents per postal code, equal to the expected number of small-scale and backyard flocks per postal code, based on the surface area of each postal code in km^2^. The model was implemented in the R programming language using the *INLA*, *spdep*, and *SpatialEpi* packages. We normalised by surface area (km^2^) rather than human population size, thereby defining a baseline assumption of equal density per unit area across postal codes. To validate the outcomes of the BYM2-model, we used self-reported estimates of neighbouring small-scale and backyard flocks within a 3 km radius of their own flock (Supplementary Fig. C.1B). Survey respondents could provide these estimates as open responses. Responses without a numeric value (e.g., “*I do not know*” or “*Many”*) were treated as missing data. In postal code regions with multiple respondents, the mean number of self-reported neighbouring small-scale and backyard flocks was used. Furthermore, numerical estimates above 1040 neighbouring flocks were removed, based on the third quartile of the median number of houses per km^2^ derived from the PC4 data.

The BYM2-model estimates and the self-reported local density measures were used to evaluate spatial coverage and identify undersampled regions. Because the two measures may exhibit non-linear associations, we assessed their concordance using a non-parametric Kendall rank correlation test. To further identify areas with potential discrepancies, we compared postal codes in the lowest quartile of model-based relative intensity with those in the highest quartile of self-reported neighbouring flock estimates (Supplementary Fig. C.1C). Postal codes exhibiting low model-based intensity despite high self-reported neighbouring flock numbers were interpreted as potentially undersampled regions.

### 2.5 Statistics: survey data analysis

The 17 survey questions relevant to this study were coded into 16 variables. New variables were created to calculate total flock sizes, the number of other pet species apart from poultry, and taxonomic bird groups based on their relevance in AIV transmission dynamics. Most questions were multiple-choice, and for some questions respondents could select multiple options or provide open-ended responses by selecting “other”, followed by a free-text specification. Free-text responses were reviewed and recoded into existing or newly defined variable categories where appropriate.

As variables did not follow a normal distribution and contained multiple outliers, descriptive statistics are presented as medians and interquartile ranges (IQRs), and associations were assessed using non-parametric tests. Data cleaning and analysis were performed in Microsoft Excel (version 2025, 16.102.25101829) and R (RStudio version 2025.09.2 + 418) using packages including *sf, ggplot2, ggspatial, forcats,* and *ggh4x*.

Missing data issues were addressed primarily through data removal. Throughout, we assumed that missingness was completely at random (MCAR) [45]. Hence, respondents with missing values were excluded only from analyses involving the affected variables rather than removed from the dataset entirely.

## 3 Results

### 3.1 Small-scale and backyard flock holding types

In total, 3660 survey respondents completed the survey. A substantial proportion of participants were recruited through the customer network of a retail chain specialised in gardening and animal care products with locations throughout the Netherlands. Out of the 3660 respondents, 135 were excluded from analyses because they did not reside in the Netherlands, kept bird species irrelevant to our study (e.g., pigeons or aviary birds), or did not fit any of the predefined holding type definitions. The remaining 3525 respondents were included in the study.

Most respondents reported keeping a backyard flock (*n* = 3336), including owners who keep poultry as pets. Smaller groups of respondents identified as petting zoo or care farm owner (*n* = 110), ornamental fowl breeder (*n* = 42), or small community farmer (*n* = 37).

### 3.2 Spatial distribution of small-scale and backyard flocks

The respondents were geographically distributed across the Netherlands (Fig. 1). Small-scale and backyard flocks were found across all urbanisation levels (Table 1). Backyard flock (47.7%) and small community farm owners (40.5%) most commonly resided in rural regions. Ornamental fowl breeders were most frequently found in suburban regions (50.0%), while petting and care farms were most commonly located in urban regions (54.5%). Overall, most respondents resided in rural regions (46.4%), with a surrounding address density below 500 addresses per km^2^.

Small-scale and backyard flocks were distributed fairly uniformly across the country. Model-based estimates of survey respondent density, based on the expected number of respondents by postal code surface size (km^2^), identified regions with relatively higher and lower intensity of respondents (Supplementary Fig. C.1A). To further assess the spatial distribution of holdings, self-reported estimates of neighbouring backyard flocks within a 3 km radius are presented in Supplementary Fig. C.1B. We compared the model-based respondent intensity with self-reported neighbouring flock estimates. A Kendall rank correlation test showed a weak positive correlation between the two measures (τ= 0.05, p = 0.004), indicating that postal code regions with higher expected respondent counts generally correspond to higher reported numbers of neighbouring flocks. A small proportion of postal codes (5.5%) showed discordance between the two measures (Supplementary Fig. C.1C). These postal codes were regions with a low model-based holding intensity but a relatively high estimate of self-reported neighbouring flocks, suggesting possible under-sampling of survey respondents in these areas.

### 3.3 Poultry owner characteristics and motivation

The median age of survey respondents was 60 years (range18–97 years) (Table 1). For petting zoos or care farms and small community farms, the median age of the poultry owner was lower (51 years) compared to owners of backyard flocks and ornamental fowl breeders (60 and 61 years, respectively). Over half of all survey respondents were female (55.8%). Respondents identifying as ornamental fowl breeders and small community farmers were, however, predominantly male (81.0% and 75.7%, respectively). Whereas most backyard flock owners (96.3%) and ornamental fowl breeders (90.5%) kept poultry on their own property at home, most respondents from petting zoos and care farms kept their poultry at a location separate from their home (69.1%). The locations mapped in Fig. 1 show the locations of the poultry flocks. A larger proportion of petting and care farm owners (77.3%), breeders (88.1%), and small community farmers (59.5%) were member of an animal organisation. In contrast, most backyard flock owners were not affiliated with any organisation (94.6%) (Table 1). Petting and care farm owners kept the greatest diversity of non-avian (pet) species, with a median of five different species per farm (range: 0–11).

Across all holding types, the most frequently reported motivations for poultry keeping were ‘as a hobby or companionship’ (95.0%), ‘for egg production’ (69.7%), and ‘for nostalgic reasons’ (12.0%) (Fig. 2). Among backyard flock owners, it was very common to keep poultry ‘as a hobby or for companionship’ (96.8%) and ‘for egg production’ (71.0%), followed by ‘nostalgic reasons’ (11.9%). Most petting and care farm owners indicated to keep poultry ‘for educational purposes’ (77.1%), followed by ‘as a hobby or companionship’ (55.0%) and ‘community feeling’ (48.6%). The top three motivations for small community farmers were ‘for egg production’ (80.6%), ‘as hobby or companionship’ (61.1%), and ‘as extra income’ (38.9%). Breeders mostly kept poultry ‘as a hobby or for companionship’ (90.5%), for ‘breeding and attending poultry exhibitions’ (83.3%), and ‘for egg production’ (50.0%). Respondents also reported additional motivations in open responses, including weed control, recycling of food scraps, as part of day support service, and for rescuing poultry from slaughter.

**Fig. 2:**
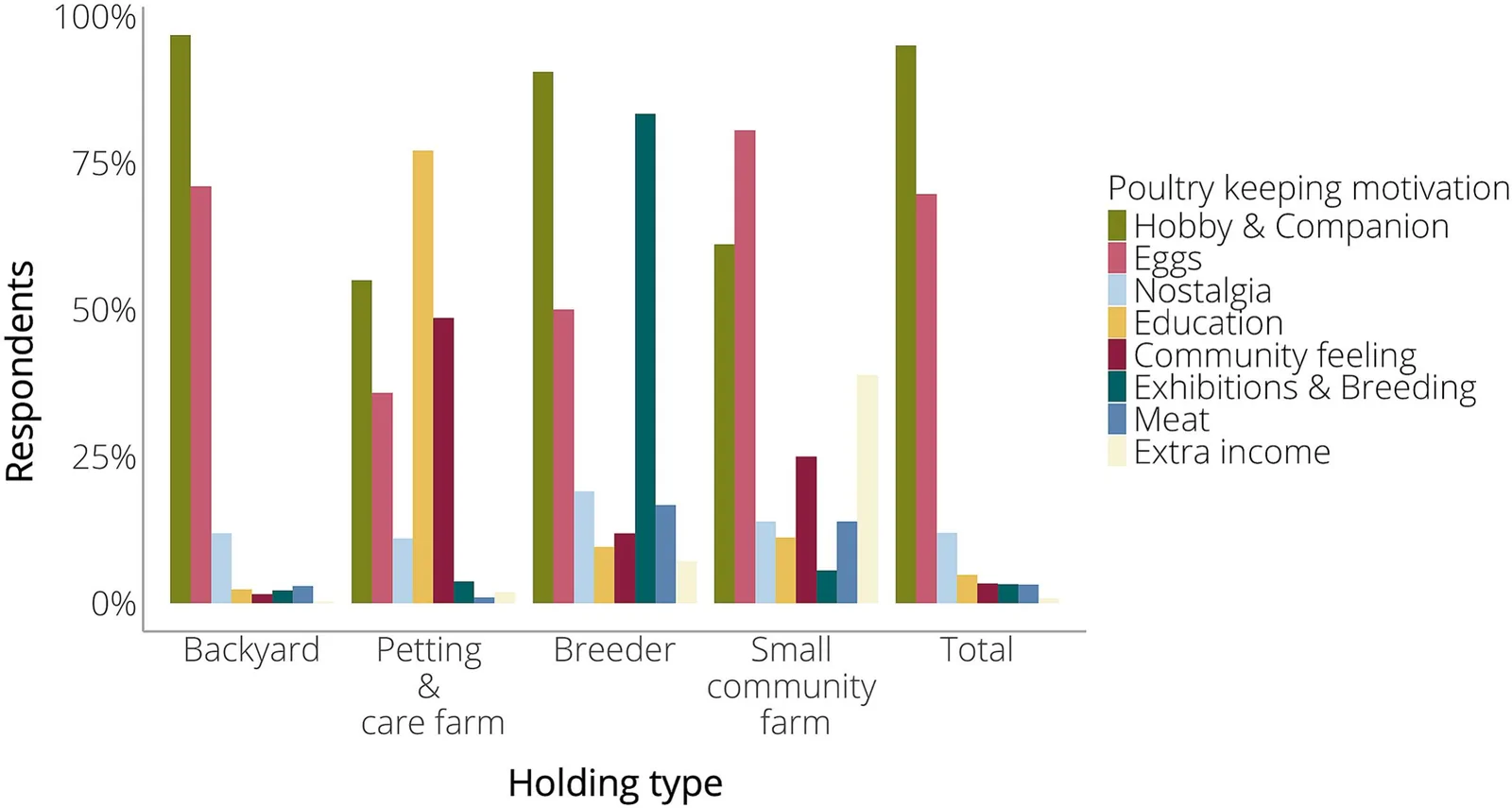
Poultry owners' motivation for poultry keeping, stratified by self-identified holding type. Bars indicate the proportion of respondents who selected a specific motivation for poultry keeping. Motivations were not mutually exclusive.

### 3.4 Flock sizes

Flock sizes differed between self-identified holding types. Backyard flock owners kept the smallest flocks, with a median flock size of 5 birds (IQR 3–8). The majority of backyard flock owners (83.8%) kept no more than ten birds (Fig. 3A). In contrast, flock sizes were larger and more variable across the other holding types. The median flock sizes kept by petting and care farms, breeders, and small community farms were 23 (IQR 12–45), 48 (IQR 26–100), and 60 (IQR 15–176), respectively. Among small community farmers, the largest proportion of respondents reported flocks of 50–249 birds (59.5%) (Fig. 3A).

**Fig. 3:**
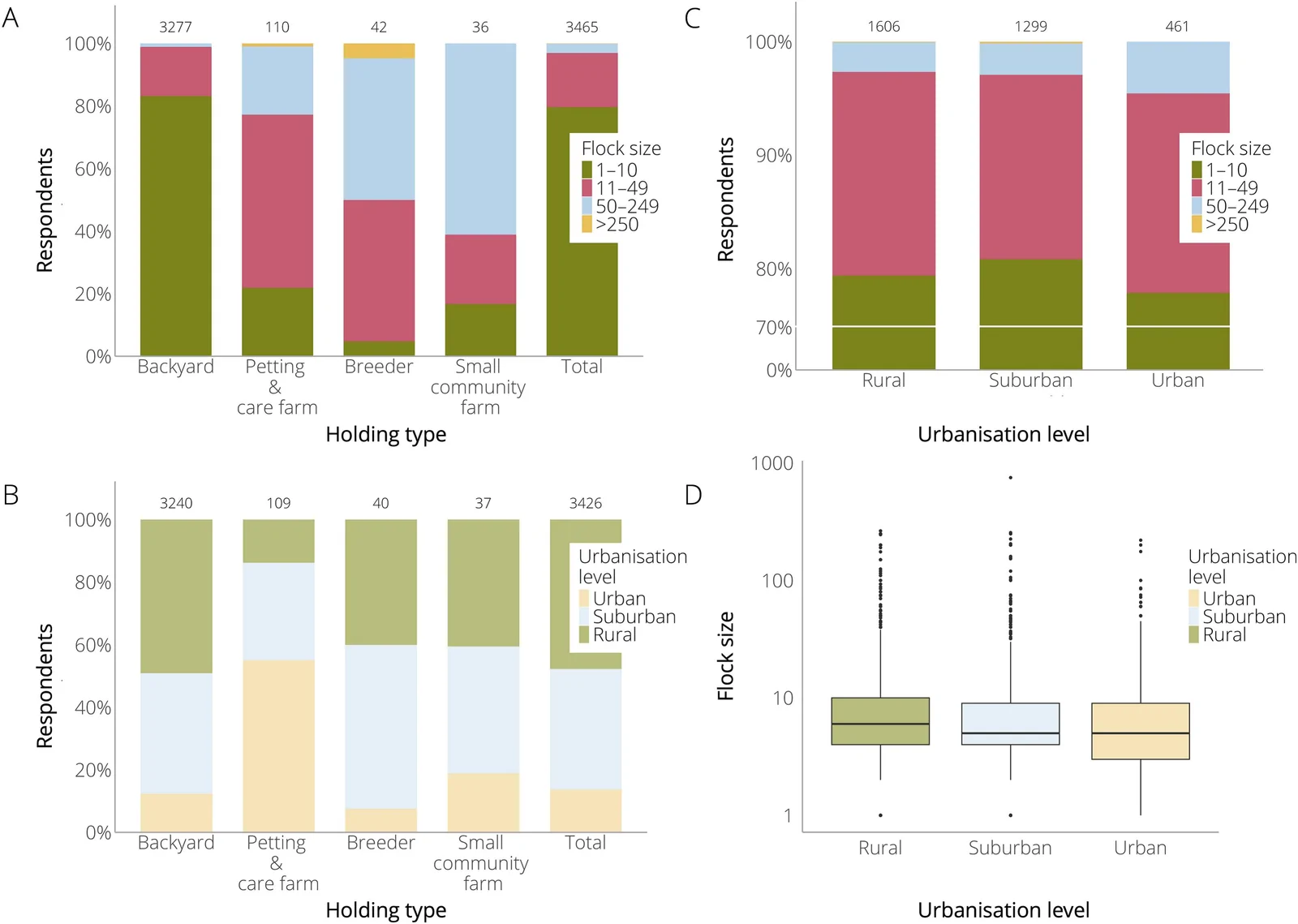
Total flock sizes in categories (1−10), (11–49), (50–249), and (≥250) by holding type and urbanisation level. The absolute number of respondents for each holding type or urbanisation level are displayed on top of the bars. A) Total flock size by holding type. B) Urbanisation level by holding type. C) Flock size by urbanisation level. D) A boxplot representing the absolute flock size by urbanisation level.

Our results showed that poultry is not kept exclusively in rural regions. Of the 3426 respondents who provided their four-digit postal code, 13.7% resided in urban areas (Fig. 3B), while most respondents resided in suburban (38.5%) or rural (47.8%) regions. Backyard flock owners most frequently resided in rural regions (47.7%), followed by suburban areas (37.4%). Ornamental fowl breeders and small community farms were also mainly found in suburban (50% and 40.5%, respectively.) and rural (38.1% and 40.5%, respectively.) regions. In contrast, the majority of petting and care farms (54.5%) was found in urban areas (Fig. 3B). Flocks kept in rural regions had a median flock size of 5 birds (IQR 4–10). For suburban regions, the median flock sizes were 5 birds (IQR 4–9) and for urban regions 5 birds (IQR 3–9). The similarity in flock sizes across urbanisation levels is visualised for flock size categories (Fig. 3C) and as absolute values (Fig. 3D). However, after excluding backyard flocks, which dominate the dataset, median flock sizes differed significantly between rural and suburban regions (Kruskal–Wallis χ^2^ = 10.734, *p* = 0.005; Dunn's test with BH correction: *p* = 0.045) and between rural and urban regions (Kruskal–Wallis χ^2^ = 10.734, *p* = 0.005; Dunn's test with BH correction: *p* = 0.003).

### 3.5 Captive bird species

The captive birds were categorised into four taxonomic bird groups based on shared biological and husbandry characteristics, as well as AIV susceptibility: chickens, other Galliformes, Anseriformes, and other bird species. Chickens were the most abundant species across all holding types, followed by Anseriformes (including ducks, geese and swans) and other non-chicken Galliformes species (e.g., turkeys, quails, guinea fowl, pheasants, and partridges) (Fig. 4A). Among all backyard flock owners, most respondents (86.7%) only kept chickens. A smaller proportion of backyard flock owners kept both Anseriformes and Galliformes (including chickens) (7.5%) or chickens and other Galliformes (3.9%). Combinations of taxonomic groups below 1% are not shown in Fig. 4. It was very rare for backyard flock owners to keep only non-chicken Galliformes (0.9%) or only captive Anseriformes (0.8%). A small proportion of backyard flock owners kept additional species, including avocets and rails, ratites, or raptors (0.2%).

**Fig. 4:**
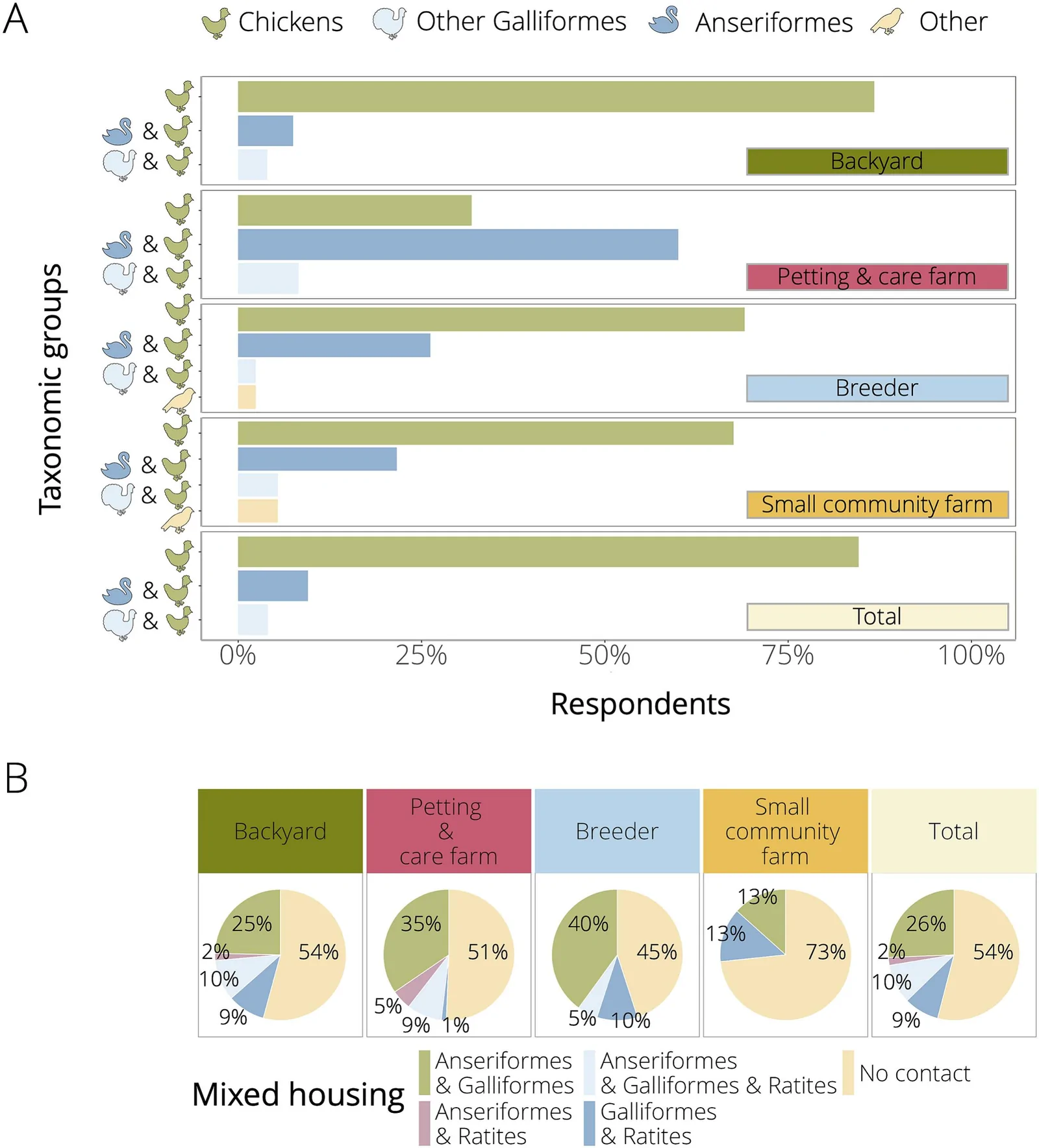
A) Taxonomic bird groups, and the combination of different taxonomic groups, kept by each holding type. Combinations of different taxonomic groups are indicated using the icons displayed in the legend at the top of the figure. B) Mixed housing or feeding practices for different taxonomic bird groups, stratified by holding type. Data are only included for respondents who reported keeping multiple taxonomic groups of captive birds.

A similar pattern was seen for breeders and small community farmers, of whom 69.1% and 67.6% respectively, only kept chickens (Fig. 4A). Among breeders (26.2%) and small community farmers (21.6%), however, keeping both Anseriformes and Galliformes was more common than among backyard flock owners. Nevertheless, most respondents kept only chickens. The majority of petting and care farms, however, kept a combination of taxonomic bird groups (68.2%) (Fig. 4A), with a substantial proportion of these holdings keeping both Anseriformes and Galliformes (including chickens) (60%). A smaller proportion kept chickens and other Galliformes species (8.2%). Only 31.8% of the petting and care farms kept only chickens, indicating that mixed-species flocks were more common among petting and care farms compared to other holding types.

For respondents who reported keeping birds from multiple taxonomic groups, the proportion using mixed housing or feeding practices is shown in Fig. 4B. Most small community farmers reported that birds from different taxonomic groups were housed or fed separately (73%), which is referred to as ‘no contact between groups’ (Fig. 4B). For the other three holding types, this proportion was around 50% (Fig. 4B). Conversely, 35% of backyard flock owners housed or fed captive Anseriformes and Galliformes (including chickens) together. For petting and care farms and breeders, this was the case for 44% and 45% of the holdings, respectively.

### 3.6 Poultry housing

The poultry owners in our data set used various indoor- and outdoor housing practices. Fig. 5 shows the most frequently mentioned combinations, as well as a category for less common combinations, stratified by self-identified holding type. Outdoor housing was classified as free-range when respondents reported to allow poultry to roam freely, even if other measures such as coops or netting were also used. The combination of an indoor closed night shelter with outdoor use of a coop or netting was most frequently reported among backyard flock owners (55.5%), petting and care farm owners (50.0%), and breeders (57.1%). Small community farmers reported the use of a mobile chicken house more frequently than the other holding types, either in combination with free-range (32.4%) or with the use of a coop or netting (13.5%). Mobile chicken houses, also referred to as ‘chicken caravans’, are movable housing units, generally combined with free-range access [5], [6]. Among small community farmers, the use of a closed night shelter combined with coops or netting was reported as frequently as mobile chicken houses combined with free-range outdoor housing (32.4%).

**Fig. 5:**
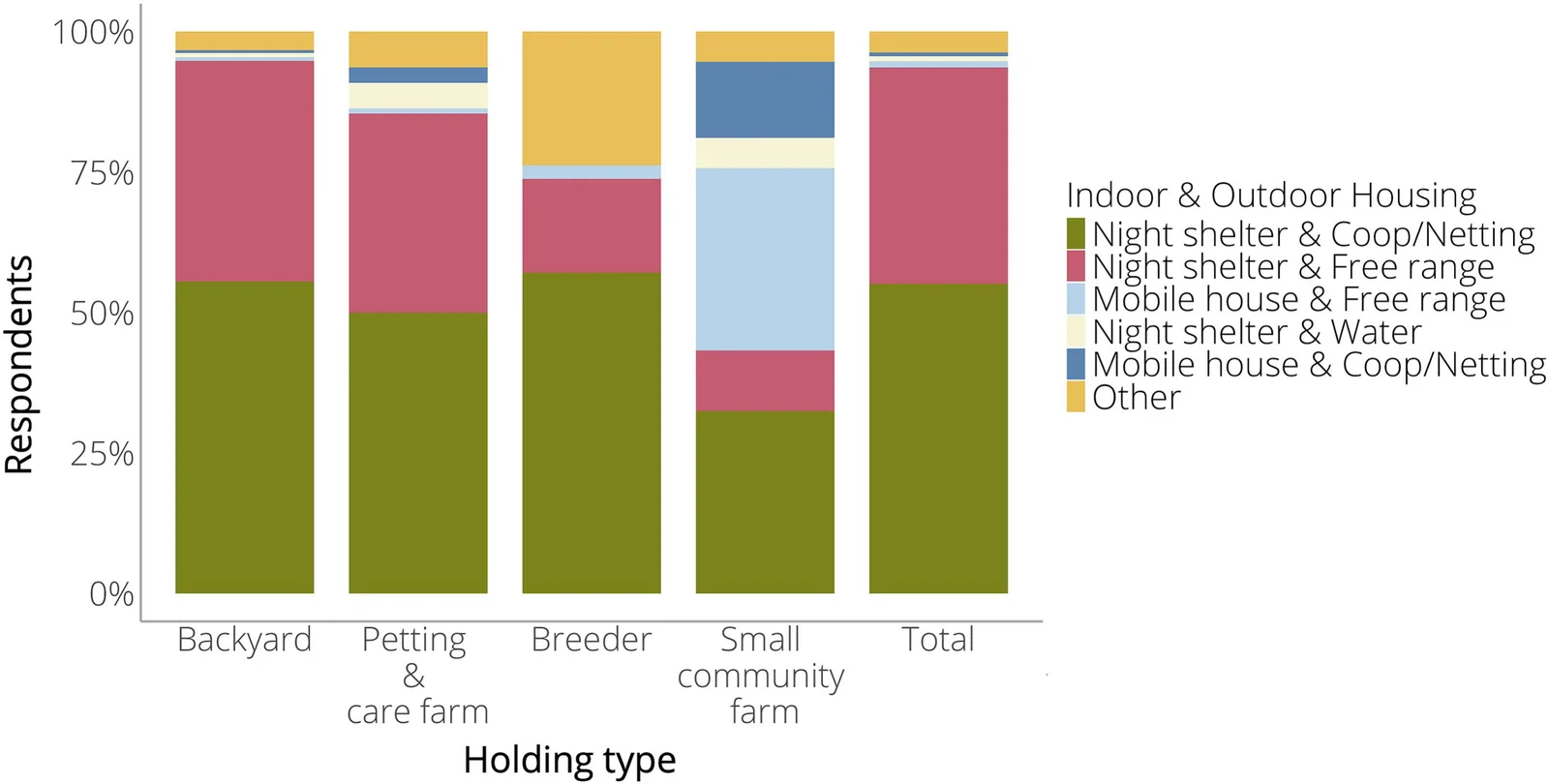
Indoor and outdoor poultry housing, stratified by self-identified holding type. The stacked bar graph shows the five most frequently reported combinations of indoor and outdoor housing.

The five most frequently reported housing combinations covered the majority of respondents among backyard flock owners, petting and care farms, as well as small community farmers. However, a substantial proportion of breeders reported less common combinations of indoor and outdoor housing (23.8%), e.g., three out of 42 breeders (7.1%) did not provide outdoor access (in combination with a closed night shelter) and two did not provide indoor housing (4.8%). Among backyard flock owners, 41 respondents did not provide indoor housing (1.2%). Of the 41 backyard flock owners who did not use indoor housing, 51.2% (0.6% of all backyard flock respondents) allowed poultry to roam freely without the use of a coop or netting. In total, 41.0% of all survey respondents (1444/3525) reported using free-range as outdoor housing, indicating that poultry were allowed to roam freely without the use of a coop or netting. Nonetheless, a small proportion of these respondents (0.6%) reported in open-text responses that they would not allow poultry to roam freely if housing orders were applied.

## 4 Discussion

The findings of this study highlight substantial variation in practices and characteristics of small-scale and backyard flocks in the Netherlands, indicating that this group represents an important and understudied component of animal health surveillance and zoonotic disease risk management. Rather than representing a single uniform sector, our results suggest that small-scale and backyard poultry keeping encompasses a spectrum of systems ranging from hobby-oriented backyard flocks to more structured, semi-commercial or educational settings. Backyard flock owners formed the largest subgroup in our study but, at the same time, kept the smallest flocks. They primarily kept flocks for hobby or companionship or egg production, whereas breeders, petting zoos and care farms, and small community farmers kept larger flocks with more function-specific motivations, e.g., breeding, exhibition, education and income generation.

Most backyard flock owners only kept chickens. This was also observed among breeders and small community farmers. Nonetheless, a substantial proportion of breeders (30.9%) and small community farms (32.4%) also reported having mixed flocks, including Anseriformes and Galliformes, indicating that multi-species flocks are relatively common across these settings. Petting and care farms, however, stood out with the greatest diversity of species overall, with Anseriformes and Galliformes (including chickens) being the most common combination, often in combination with non-avian species. Such multi-species flocks may increase opportunities for indirect pathogen transmission through shared environments, equipment, or human-mediated contact, and non-avian animals may act as mechanical carriers of contaminated material between indoor and outdoor spaces, as described for rodents or wild, terrestrial mammals [45], [46]. In addition, co-housing or close contact between different poultry taxonomic groups was frequently reported across holding types with multiple poultry species. The majority of small community farmers housed and fed birds from different taxonomic groups separately. In contrast, approximately half of the petting zoos and care farms, ornamental fowl breeders, as well as the few mixed backyard flocks, housed and fed birds from different taxonomic groups together. For the specific case of AIVs, Anseriformes, such as ducks, geese, and swans, are the main reservoir hosts and can act as asymptomatic carriers, shedding large amounts of the virus through faeces, saliva, or nasal secretions [27], [36]. Housing and feeding Anseriformes and Galliformes together may therefore increase the risk of introducing the virus into chicken flocks, potentially resulting in high mortality due to HPAI infection.

Flock composition and housing practices in our study varied across holding types, due to differences in poultry keepers' motivations, as also reported in prior research [34]. Housing practices generally reflected a balance between protection and outdoor access. Across all holding types, poultry owners most often combined an indoor closed night shelter with outdoor use of a coop or netting to house their flock, but a substantial proportion of respondents also reported free-range housing. Outdoor access may increase exposure to wild waterfowl and other wildlife, which are known reservoirs of AIVs, and are recognised as key sources of pathogen introduction into, for example, large-scale commercial poultry farms through direct or indirect contact [49], [50], [51], [52], [53].

A recent modelling study on the impact of outdoor farming, farm size, and farm density on HPAI epidemics in the Netherlands showed that more outdoor farming could result in an increased simulated epidemic size (number of infected farms) [53]. Reducing farm size, however, resulted in smaller simulated epidemics than corresponding scenarios without size reduction, although this did not fully offset the increase associated more outdoor farming. This study focused on commercial poultry farms, and therefore, its findings cannot be directly extrapolated to small-scale and backyard flock settings, which can differ substantially in flock size and management.

Nevertheless, the underlying concern that outdoor access may increase opportunities for contact with wild birds is also relevant to small-scale and backyard flocks, although direct evidence quantifying this risk in these systems remains limited. The risk of spillover events from wildlife to backyard poultry likely depends on many factors, including biosecurity practices and the level and frequency of wild bird-poultry contact [54], [55]. These factors, which play an important role in assessments of risks for small-scale and backyard poultry holdings, are currently under study (Oosters, et al.*,* unpublished results).

We studied the characteristics and practices of small-scale and backyard poultry flock owners using a cross-sectional study design. Because recruitment relied on voluntary participation and non-probability sampling, the sampling method may have introduced bias [56], [57], including potential spatial bias in participation. Consequently, our sample cannot be considered strictly representative of the national population of backyard poultry owners. However, considering the inclusion of 3500 survey respondents, while the number of backyard poultry owners was estimated at approximately 100,000 in 2004 [30], our findings were likely based on a substantial proportion of this population (∼3.5%), providing valuable descriptive insight into a sector for which structured data are currently scarce.

Visual inspection of the raw location data based on postal code centroids, and the model-based respondent intensities (Supplementary Fig. C.1 A) suggested that small-scale and backyard flocks were distributed fairly uniformly across the Netherlands. Respondents were more frequently located in suburban and rural areas compared with the general population. A similar pattern, with more backyard flocks in suburban and rural regions compared to urban areas, has been reported in France and New Zealand [1], [35]. While housing constraints may partly explain the lower number of flocks in urban areas, the shift in poultry keeping motivations and corresponding small flock sizes observed in our study, with median flock sizes below 60 birds, may help explain why a considerable proportion (13%) were still located in urban areas. While the overall spatial coverage was broad, some regional undersampling likely occurred, potentially reflecting the recruitment and survey distribution process, rather than true absence of holdings. The number of store locations, of a retail chain that contributed to survey dissemination, was limited in some regions because of presence of a competing chain, which may explain some of the local undersampling [58].

This study provides a detailed overview of a sector that is currently underrepresented in health monitoring, surveillance, and policy. The marked variation in characteristics and management practices between the different small-scale and backyard flock holding types, as well as in comparison to other poultry sectors, highlights the importance of health surveillance and zoonotic risk management in these small-scale holdings. As the Netherlands is known for its high human population density, recurrent AIV outbreaks in large-scale commercial poultry, as well as its role as an important breeding and stopover site for waterbird populations migrating along the East Atlantic Flyway [59], it is a particularly relevant setting to study emerging and re-emerging zoonotic pathogens at the wildlife-domestic animal-human interface. While these contextual factors make the Netherlands a particularly informative case study, the insights gained from this study are likely applicable to similar small-scale and backyard poultry sectors beyond the Dutch context, particularly where comparable wildlife-domestic animal-human interfaces occur.

## 5 Conclusion

This study provides a first nationwide characterisation of small-scale and backyard poultry holdings in the Netherlands and shows how this diverse and previously undercharacterised sector operates at the wildlife–domestic animal–human interface. The data provide a foundation for more explicitly including these holdings in One Health surveillance and risk assessment. Future research should focus on identifying and quantifying risk factors for pathogen spillover, including wild bird–poultry contact and biosecurity practices applied by owners, to better inform risk-based surveillance, prevention and targeted policy interventions.

## Declaration of generative AI and AI-assisted technologies in the manuscript preparation process

During the preparation of this work the authors used the OpenAI tool ChatGPT to assist with writing and improving codes for programming in *R*, as well as to refine written text*.* After using this tool/service, the authors reviewed and edited the content as needed and take full responsibility for the content of the published article.

## CRediT authorship contribution statement

**J.J. Oosters:** Writing – review & editing, Writing – original draft, Visualization, Validation, Methodology, Investigation, Formal analysis, Data curation. **M. van Boven:** Writing – review & editing, Validation, Supervision, Project administration, Methodology, Funding acquisition, Conceptualization. **M.J.M. Bonten:** Writing – review & editing. **J.A. Stegeman:** Writing – review & editing, Funding acquisition, Conceptualization. **F.C. Velkers:** Writing – review & editing, Validation, Supervision, Resources, Project administration, Methodology, Funding acquisition, Conceptualization.

## Ethics approval

As part of this study, data were collected from Dutch small-scale and backyard poultry owners through an online, nationwide survey. Formal ethical approval was not required for this questionnaire study under the applicable privacy and data-protection legislation and institutional requirements. Information about the study, data use, and privacy, which were discussed and agreed upon with an Utrecht University privacy officer in accordance with Utrecht University regulations, was provided to study participants before they were able to access and answer survey questions. Informed consent was obtained from all survey respondents upon accepting these conditions by ticking a box confirming that they had read the provided information.

## Funding

This work was supported by the 10.13039/501100001826Netherlands Organisation for Health Research and Development (ZonMw) under grant number 10150022310017.

## Declaration of competing interest

The authors declare the following financial interests/personal relationships which may be considered as potential competing interests: Michiel van Boven reports financial support was provided by Netherlands Organisation for Health Research and Development. If there are other authors, they declare that they have no known competing financial interests or personal relationships that could have appeared to influence the work reported in this paper.

## Data Availability

The data that has been used is confidential.
