# Supplementary material for: Mapping the landscape of small-scale and backyard poultry flocks in the Netherlands: a nationwide survey for One Health surveillance
Source: One Health. 2026 Oct 23;23:101626. doi: 10.1016/j.onehlt.2026.101626 (PMC13763572; doi:10.1016/j.onehlt.2026.101626)
Supplement: Supplementary file 1 [file mmc1.pdf]

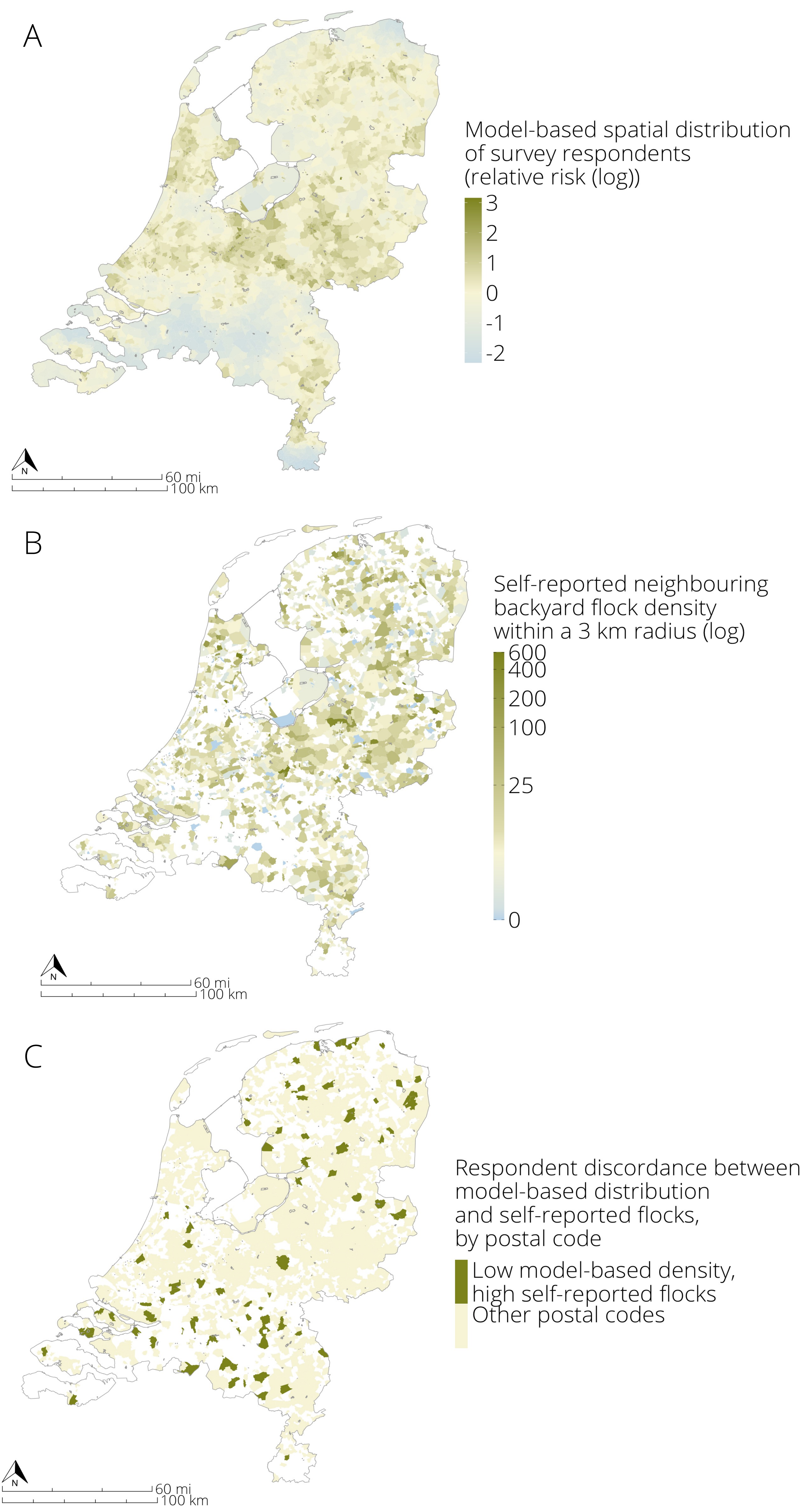
A
Model-based spatial distribution of survey respondents (relative risk (log))
3
2
1
0
-1
-2
N
60 mi
100 km
B
Self-reported neighbouring backyard flock density within a 3 km radius (log)
600
400
200
100
25
0
N
60 mi
100 km
C
Respondent discordance between model-based distribution and self-reported flocks, by postal code
Low model-based density, high self-reported flocks
Other postal codes
N
60 mi
100 km
