# Supplementary material for: Mapping the landscape of small-scale and backyard poultry flocks in the Netherlands: a nationwide survey for One Health surveillance
Source: One Health. 2026 Oct 23;23:101626. doi: 10.1016/j.onehlt.2026.101626 (PMC13763572; doi:10.1016/j.onehlt.2026.101626)
Supplement: Supplementary file 2 [file mmc2.docx]

### Manuscript: “Mapping the landscape of small-scale and backyard poultry flocks in the Netherlands: a nationwide survey for One Health surveillance”

Supplementary File A: Questionnaire

This appendix includes a copy of the questionnaire, which was distributed and created online using Qualtrics (Provo, UT). The questionnaire was developed in Dutch and translated to English for the purpose of this publication. Questions were divided into six different themes, as presented below.

1. Theme 1: Poultry owner characteristics
   I. Information about the poultry owner
   II. Information about the household
   III. Information about the poultry holding
2. Theme 2: Flock characteristics
   I. Information about the flock composition and flock size
3. Theme 3: Housing and husbandry practices
   I. Information about housing and care taking of poultry
   II. Information about the immediate surroundings
   III. Information about other companion animals
4. Theme 4: Poultry-wildlife interactions
   I. Information about wild bird-visits
   II. Information about dead wild birds
   III. Information about pests and predators
5. Theme 5: Poultry-human contact
   I. Information about the level of contact
   II. Information about the disposal of manure and litter
   III. Information about deceased poultry
   IV. Information about the contact between the poultry and other people
   V. Information about contact with other poultry holdings
   VI. Information about the sale of poultry products
6. Theme 6: Avian influenza and mitigation measures
   I. Information about bird flu and mitigation measures
   II. Information about poultry vaccination
   III. Information about previous bird flu infections
   IV. Information about the seasonal flu vaccination status of the poultry owner
7. Final questions

# Questionnaire: Avian influenza and Backyard Flocks

### Introduction to the study, explanation of the research topic, and informed consent

**Introduction** Thank you for filling out our questionnaire. We are investigating whether, and how, avian influenza can spread from wild birds to poultry and from poultry to humans. In this questionnaire, the term “poultry” refers to the birds you keep, such as gallinaceous birds (for example chickens, pheasants and quail), waterfowl (ducks, geese and swans) and ratites (such as rheas and emus).

**More information about the study** Would you like more information about the study? Please read the document Information about the study: avian influenza in hobby poultry. If you have questions about the survey study, you can contact the researcher Jolyn Oosters ([j.j.oosters@uu.nl](mailto:j.j.oosters@uu.nl)). If you have questions about how Utrecht University handles your personal data, you can consult our privacy statement or contact [privacy-dgk@uu.nl](mailto:privacy-dgk@uu.nl).

**Would you like to participate in the study?**

1. Yes, I have read and understood the information above, and I give my consent to participate in this study. (1)
2. No, I do not wish to participate in this study. Take me to the end of the questionnaire. (2)

*Skip To: End of Questionnaire If Would you like to participate in the study?  = No, I do not wish to participate in this study. Take me to the end of the questionnaire.*

### **Theme 1: Poultry owner characteristics**

1.1 Information about the poultry owner
**General information about yourself**. We use this information only for anonymised research.

Q1 What is your age?

_____________________________

Q2 Which gender do you identify with?

o Male (1)
o Female (2)
o Other, namely: (3) ________________________________________________
o Prefer not to say (4)

Q3 What is the highest level of education you have completed?

o No formal education (1)
o Primary education, VMBO, MBO1 (2)
o HAVO, VWO, MBO2–4 (3)
o HBO (University of Applied Sciences), WO (Research university) (4)
o Other, namely: (5) ________________________________________________

Q4 In which country do you live, or where do you keep your poultry?

o The Netherlands (1)
o Belgium (2)
o Other (3)

*Skip To: End of Questionnaire If In which country do you live, or where do you keep your poultry? = Other (3)*

*Display this question:
If In which country do you live, or where do you keep your poultry? = The Netherlands (1)*

Q5 What is the postal code (NL) of the location where you keep your poultry?

*We use postal codes to gain an overview of the distribution of poultry holdings. This information is used only for anonymised research. If you are willing to share the postal code for research purposes, you may enter it in the following formats: 1234AB or* *1234.*

_____________________________

*Display this question:
If In which country do you live, or where do you keep your poultry? = Belgium (2)*

Q6 What is the postal code (BE) of the location where you keep your poultry?

*We use postal codes to gain an overview of the distribution of poultry holdings. This information is used only for anonymised research. If you are willing to share the postal code for research purposes, you may enter it in the following format:* *1234.*

_____________________________


1.2 Information about the household
**Information about your household*.*** We use this information only for anonymised research.

Q7 What are the ages of everyone in your household (including yourself)?

*Example of a household with four people: 50, 45, 15, 12*

_____________________________

Q8 What are the ages of the people in your household who have direct contact with the poultry (including yourself)?

*By “direct contact” we mean caring for the poultry, such as feeding them, collecting eggs, cleaning the enclosure, as well as picking up, petting, or cuddling the birds.

Example of a household where three people have direct contact with the poultry: 50, 45, 15.*

_____________________________

1.3 Information about the poultry holding

Q9 Do you keep the poultry on the same property as your home?

o Yes (1)
o No (2)

Q10 In what type of landscape do you keep the poultry?

o Agricultural rural area (farms, meadows, etc.) (1)
o Natural rural area (forests, heathland, etc.) (2)
o Wadden or coastal area (3)
o Suburban area: village, suburb, or edge of a town/city (4)
o Urban area (5)
o Other, namely: (6) ________________________________________________

Q11 Do you keep the poultry in a water-rich area?

*Water-rich areas include, for example, polders, lakes, and river areas where there is a lot of water. Areas such as sandy soils, forests, and hilly areas are often not water-rich.*

o Yes (1)
o No (2)

Q12 Which description best fits your poultry holding?

o Hobby holding (including pets) (1)
o Small-scale commercial holding (fewer than 250 birds) (2)
o Breeder or trader of (ornamental) poultry (3)
o Petting zoo (4)
o Care farm (5)
o Other, namely: (6) ________________________________________________

Q13 Are you affiliated with an organisation or association?
 *You may select multiple answers for this question.*

▢ No (1)
▢ Aviornis (2)
▢ Kleindier Liefhebbers Nederland (KLN) (3)
▢ KleindierNed (4)
▢ Netherlands Federation of Aviculture Societies (NBvV) (5)
▢ Herenboeren (6)
▢ Caring Farmers (7)
▢ GeluksVogel (8)
▢ Association of Urban and Petting Farms (vSKBN) (9)
▢ Other, namely: (10) ________________________________________________

Q14 Why do you like keeping poultry?

*You may select multiple answers for this question.*

▢ Hobby (1)
▢ Companionship (2)
▢ Eggs (3)
▢ Meat (4)
▢ Additional income (5)
▢ Educational purposes (6)
▢ Research (7)
▢ Exhibitions (8)
▢ Connection with the community (9)
▢ Nostalgia (10)
▢ Other, namely: (11) ________________________________________________

### **Theme 2: Flock characteristics**

### 2.1 Information about the flock composition and flock size

Q15 Which types of poultry did you keep in the past year?

*In this questionnaire, we distinguish three groups: gallinaceous birds, waterfowl, and ratites. Gallinaceous birds include, for example, chickens, turkeys, pheasants, peacocks and quail. Waterfowl include ducks, geese and swans. Ratites include rheas and emus.*

You may select multiple answers for this question.

▢ Chickens (1)
▢ Turkeys (2)
▢ Pheasants (3)
▢ Peacocks (4)
▢ Quail (12)
▢ Ducks (5)
▢ Geese (6)
▢ Swans (7)
▢ Ratites (10)
▢ Other, namely: (11) ________________________________________________

*Display this question:*

*If Which types of poultry did you keep in the past year?* ***=*** *Chickens (1)*

*Or Which types of poultry did you keep in the past year?* *= Turkeys (2)*

*Or Which types of poultry did you keep in the past year?* ***=***  *Pheasants (3)*

*Or Which types of poultry did you keep in the past year?* ***=*** *Peacocks (4)*

*Or Which types of poultry did you keep in the past year?* ***=*** *Quails (12)*

*Or Which types of poultry did you keep in the past year?* ***=*** *Other, namely (11)* *Text Response Is Not Empty*

Q16 How many gallinaceous birds did you have on average in the past year?

*Gallinaceous birds include, among others, chickens, turkeys, pheasants, peacocks and quail.
If you did not have any gallinaceous birds, you may enter 0.*

_____________________________

*Display this question:*

*If Which types of poultry did you keep in the past year?* ***=*** *Ducks (5)*

*Or Which types of poultry did you keep in the past year?* ***=*** *Geese (6)*

*Or Which types of poultry did you keep in the past year?* *= Swans (7)*

*Or Which types of poultry did you keep in the past year? = Other, namely (11)* *Text Response Is Not Empty*

Q17 How many captive waterfowl did you have on average in the past year?

*Waterfowl include, among others, ducks, geese and swans. If you did not have any waterfowl, you may enter 0.*

_____________________________

*Display this question:*

*If Which types of poultry did you keep in the past year?* *= Ratites (10)*

*Or Which types of poultry did you keep in the past year?* ***=*** *Other, namely (11****)*** *Text Response Is Not Empty*

Q18 How many ratites did you have on average in the past year?

*Ratites include, among others, rheas and emus. If you did not have any ratites, you may enter 0.*

_____________________________

Q19 Do your gallinaceous birds, waterfowl or ratites have direct contact with each other?

*For example, through sharing the same enclosure or sharing drinking water or feed. Examples of waterfowl include ducks, geese and swans. Gallinaceous birds include chickens, and ratites include, among others, emus.*

o No, they do not have contact with each other (1)
o No, I only have one group (2)
o Yes, gallinaceous birds, waterfowl and ratites (3)
o Yes, gallinaceous birds and waterfowl (4)
o Yes, gallinaceous birds and ratites (5)
o Yes, waterfowl and ratites (6)

### **Theme 3: Housing and husbandry practices**

3.1 Information about housing and care taking of poultry

Q20 What do you use as indoor housing for your poultry?

o A stable (1)
o A shed (2)
o A night coop (3)
o A mobile chicken coop (4)
o Other, namely: (5) ________________________________________________
o The poultry does not have indoor housing (6)

Q21 What do you use as outdoor housing for your poultry?

o An enclosed run or pen (1)
o An open roaming area without enclosure (2)
o A large fenced area or pasture (3)
o A pond or water area with surrounding fencing (4)
o Other, namely: (5) ________________________________________________
o The poultry does not have outdoor housing (6)

Q22 What type of water does the poultry drink?
*You may select multiple answers for this question.*

▢ Tap water (1)
▢ Ditch water (2)
▢ Collected rainwater (3)
▢ Other, namely: (4) ________________________________________________

Q23 Can wild birds, pests, or predators access the rain barrel, ditch, or another water source used for the poultry?

*This question does not apply to tap water.
Wild birds are birds that live freely in nature. By “pests” and “predators” we mean, for example, rats and foxes.*

o Yes (1)
o No (2)
o I only use tap water as a water source (3)

### 3.2 Information about the immediate surroundings

Q24 How far is your poultry from the nearest natural water source, such as a ditch or river?

o Less than 500 metres (1)
o 500 to 1000 metres (2)
o 1000 to 1500 metres (3)
o More than 1500 metres (4)

Q25 How many other small-scale or hobby poultry holdings are there within 3 km of your holding?
 *You may provide an estimate for this question.*

_____________________________

### 3.3 Information about other companion animals

Q26 In the past year, did you have other farm animals or companion animals besides poultry?
*You may select multiple answers for this question.*

▢ No (1)
▢ Yes, cattle (2)
▢ Yes, pigs (3)
▢ Yes, horses (4)
▢ Yes, sheep (5)
▢ Yes, goats (6)
▢ Yes, dog(s) (7)
▢ Yes, cat(s) (8)
▢ Other, namely: (9) ________________________________________________

*Display this question:*

*If In the past year, did you have other farm animals or companion animals besides poultry?* *!= No (1)*

Q27 Do the other (farm) animals have contact with the poultry?

*By “contact” we mean that the other animals and the poultry move freely among each other.
You may select multiple answers for this question.*

▢ No (1)
▢ Yes, cattle (2)
▢ Yes, pigs (3)
▢ Yes, horses (4)
▢ Yes, sheep (5)
▢ Yes, goats (6)
▢ Yes, dog(s) (7)
▢ Yes, cat(s) (8)
▢ Other, namely: (9) ________________________________________________

*Display this question:*

*If In the past year, did you have other farm animals or companion animals besides poultry?* *!= No (1)*

Q28 Can the other (farm) animals have contact with wild birds?

*For example, by grazing or moving in areas where wild birds are present.
You may select multiple answers for this question.*

▢ No (1)
▢ Yes, cattle (2)
▢ Yes, pigs (3)
▢ Yes, horses (4)
▢ Yes, sheep (5)
▢ Yes, goats (6)
▢ Yes, dog(s) (7)
▢ Yes, cat(s) (8)
▢ Other, namely: (9) ________________________________________________

### **Theme 4: Poultry-wildlife interactions**

### 4.1 Information about wild bird-visits

Q29 Which wild birds did you see within a radius of 100 metres of the poultry in the past year?

*Wild birds are birds that live freely in nature and are not kept by people. Examples include wild waterfowl such as ducks, geese and swans. Sparrows, crows and birds of prey are also examples of wild birds.*

*You may select multiple answers for this question.*

▢ None (1)
▢ Gulls or other coastal birds (2)
▢ Waterfowl, such as ducks, geese or swans (3)
▢ Moorhens or coots (9)
▢ Wild pheasants or partridges (10)
▢ Birds of prey (4)
▢ Crows or corvids (5)
▢ Herons, storks or cranes (6)
▢ Pigeons or related species (7)
▢ Small songbirds, such as blackbirds and starlings, or swallows (8)
▢ Other, namely: (11) ________________________

*Display this question:*

*If Which wild birds did you see within a radius of 100 metres of the poultry in the past year?*
 *!= None (1)*

Q30 Which wild birds had contact with the poultry in the past year?

*By “contact”* *we mean that wild birds and poultry move freely among each other. It may also mean that wild birds visit the area where the poultry stay when the poultry are indoors and leave droppings there. Sometimes wild birds drink from or eat the poultry’s water or feed.*

*You may select multiple answers for this question.*

▢ None (1)
▢ Gulls or other coastal birds (2)
▢ Waterfowl, such as ducks, geese or swans (3)
▢ Moorhens or coots (9)
▢ Wild pheasants or partridges (10)
▢ Birds of prey (4)
▢ Crows or corvids (5)
▢ Herons, storks or cranes (6)
▢ Pigeons or related species (7)
▢ Small songbirds, such as blackbirds and starlings, or swallows (8)
▢ Other, namely: (11) ________________________________________________

*Display this question:*

*If Which types of poultry did you keep in the past year? = Ducks*

*Or Which types of poultry did you keep in the past year? = Geese*

*Or Which types of poultry did you keep in the past year? = Swans*

*Or Which types of poultry did you keep in the past year? = Other, namely (11) Text Response Is Not Empty*

*And If Which wild birds did you see within a radius of 100 metres of the poultry in the past year?
 != None (1)*

Q31 In the past year, have you seen wild waterfowl in the water where your captive waterfowl swim?

*By “captive waterfowl” we mean the waterfowl you keep as poultry. “Wild waterfowl” are, for example, ducks, geese or swans that are not kept by you as poultry.*

*You may select multiple answers for this question.*

▢ No (1)
▢ Yes, gulls or other coastal birds (2)
▢ Yes, waterfowl such as ducks, geese or swans (3)
▢ Yes, moorhens or coots (5)
▢ Yes, herons, storks or cranes (6)
▢ My waterfowl do not swim in water (8)
▢ I do not have waterfowl (9)
▢ Other, namely: (7) ________________________________________________

### 4.2 Information about dead wild birds

Q32 Do you know whether dead wild birds were found in the past year?

o I do not know (1)
o Yes, on my own property (2)
o Yes, not on my property but nearby (3)
o Yes, both on my property and nearby (4)
o No dead wild birds were found (5)

### 4.3 Information about pests and predators

Q33 Do you think that pests or predators, such as rats or foxes, had contact with the poultry in the past year?

*By “contact” we mean that you see pests or predators among the poultry, or near the feed or water. This may also occur at night, for example if you notice packaging that has been gnawed open.*

*You may select multiple answers for this question.*

▢ No (1)
▢ Yes, pests such as rats or mice (2)
▢ Yes, predators such as foxes or martens (3)
▢ Other, namely: (4) __________________________

### **Theme 5: Poultry-human contact**

### 5.1 Information about the level of contact

Q34 Do you or others who care for the poultry touch the animals, for example by picking them up, cuddling or petting them?

o Often (2)
o Sometimes (3)
o Rarely (4)
o Never (5)

*Display this question:*

*If Do you or others who care for the poultry touch the animals, for example by picking them up, cuddling or petting them? != Never (5)*

Q35 Do you (or others) wash your hands after contact with the poultry?

*For example, after picking up, cuddling or petting poultry, but also after collecting eggs or cleaning the poultry enclosure.*

o Always (1)
o Often (2)
o Sometimes (3)
o Rarely (4)
o Never (5)
o I do not have direct contact with the poultry (6)

*Display this question:*

*If Do you or others who care for the poultry touch the animals, for example by picking them up, cuddling or petting them? != Never (5)*

Q36 Do you (or others) wear protective equipment when you have contact with the poultry?

*You may select multiple answers for this question.*

▢ No (1)
▢ Yes, gloves (2)
▢ Yes, different footwear (3)
▢ Yes, different clothing (4)
▢ Yes, a face mask (5)
▢ Other, namely: (6) ________________________________________________

### 5.2 Information about the disposal of manure and litter

Q37 What do you do with used litter and poultry manure?

*You may select multiple answers for this question.*

▢ Dispose of it in the grey or green wheelie bin (1)
▢ Compost it (2)
▢ Use it as fertiliser or ground cover on your own land (3)
▢ Use it as fertiliser or ground cover elsewhere (4)
▢ Dispose of it via the sewer through a drain on the property (5)
▢ Professional manure processing (6)
▢ Other, namely: (7) ________________________________________________

### 5.3 Information about deceased poultry

Q38 What do you do with dead poultry?

*This refers to poultry that has died unexpectedly, not poultry that has been slaughtered, for example.

You may select multiple answers for this question.*

▢ Bury it on your own property (2)
▢ Dispose of it in the grey wheelie bin (10)
▢ Dispose of it in the green wheelie bin (3)
▢ Collection by the municipality (4)
▢ Take it to a veterinarian (5)
▢ Professional carcass disposal service (6)
▢ Feed it to other animals (7)
▢ For own consumption (8)
▢ No poultry has died yet (1)
▢ Other, namely: (9) ________________________________________________

*Display this question:*

*If What do you do with dead poultry? != No poultry has died yet (1)*

Q39 Do you use protective equipment when handling dead poultry (and possibly dead wild birds)?
 *You may select multiple answers for this question.*

▢ No (1)
▢ Yes, gloves (2)
▢ Yes, washing hands (3)
▢ Yes, different clothing (4)
▢ Yes, a face mask (5)
▢ Other, namely: (6) ________________________________________________

### 5.4 Information about the contact between the poultry and other people

Q40 In the past year, have people from outside your household had contact with the poultry?

*You may select multiple answers for this question.*

▢ No (1)
▢ Yes, as a caretaker of the poultry (employee or volunteer) (3)
▢ Yes, as part of care or day activities (4)
▢ Yes, visitors to the holding (5)
▢ Other, namely: (6) ________________________________________________

### 5.5 Information about contact with other poultry holdings

Q41 In the past year, have you or someone else who cares for your poultry had contact with poultry from another holding?

*For example, poultry from another backyard flock, a petting zoo, or from a commercial poultry farm.*

o Yes (1)
o No (2)

*Display this question:*

*If In the past year, have you or someone else who cares for your poultry had contact with poultry from another holding? = Yes (1)*

Q42 With poultry from which type of holding did you (or others) have contact, besides your own poultry?

*You may select multiple answers for this question.*

▢ A large-scale commercial holding (1)
▢ A small-scale commercial holding (fewer than 250 birds) (2)
▢ A backyard flock (including pets) (3)
▢ An (ornamental) poultry breeder (4)
▢ A petting zoo (5)
▢ A care farm (6)
▢ Other, namely: (7) ________________________________________________

*Display this question:*

*If In the past year, have you or someone else who cares for your poultry had contact with poultry from another holding? = Yes (1)*

Q43 Do you (or others) use protective equipment when in contact with other poultry?

*You may select multiple answers for this question.*

▢ No (1)
▢ Yes, gloves (2)
▢ Yes, different footwear (3)
▢ Yes, different clothing (4)
▢ Yes, a face mask (5)
▢ Yes, washing hands (6)
▢ Other, namely: (7) ________________________________________________

### 5.6 Information about the sale of poultry products

Q44 In the past year, did you sell or give away animal products from your poultry?

*By “animal products” we mean, for example, eggs and meat.

You may select multiple answers for this question.*

▢ No (1)
▢ Yes, customers regularly come onto the property (2)
▢ Yes, but customers do not come onto the property (for example, sales via a roadside box) (3)
▢ Yes, I sell to shops, markets or other outlets (5)
▢ Other, namely: (4) ________________________________________________

### **Theme 6: Avian influenza and mitigation measures**

### 6.1 Information about bird flu and mitigation measures

Q45 How likely do you think it is that your poultry will become infected with avian influenza?

*For this question, you may give an estimate using the scale below. The number 1 means “very small chance” and the number 5 means “very high chance”.*

|  | 1 | 2 | 3 | 4 | 5 | I do not know |
| --- | --- | --- | --- | --- | --- | --- |
| Risk of bird flu infection | ○ | ○ | ○ | ○ | ○ | ○ |
|  |  |  |  |  |  |  |

Q46 Have you taken measures to reduce contact between wild birds and your poultry?

*You may select multiple answers for this question.*

▢ No (1)
▢ Yes, by screening the outdoor run or enclosure with mesh/netting (2)
▢ Yes, by covering the swimming water of kept waterfowl (3)
▢ Yes, by covering feed, roughage, water and bedding (4)
▢ Yes, my poultry only has indoor housing (5)
▢ I am not aware of the risk of contact between wild birds and poultry (6)
▢ Other, namely: (7) ________________________________________________

Q47 Are you able to comply with a housing order or a screening requirement?

*In the Netherlands, a housing order or the mandatory use of netting may sometimes apply when there is an increased risk of avian influenza outbreaks.*

*Housing order: Poultry must remain indoors in an enclosed space, such as a stable, barn or shed. This is to completely prevent contact with wild birds.*

*Mandatory use of netting: Contact between poultry and possibly infected wild birds is reduced by shielding the poultry enclosure with mesh or by keeping the birds in an aviary.*

o Neither measure is possible (1)
o Both measures are possible (2)
o Only the use of netting is possible (3)
o Only a housing order is possible (4)
o Other, namely: (5) ________________________________________________

### 6.2 Information about poultry vaccination

Q48 What would be reasons for you to have your poultry vaccinated against avian influenza, if this were possible?

*You may select multiple answers for this question.*

▢ No reasons to vaccinate (1)
▢ Protection of poultry against avian influenza (2)
▢ Protection of other kept animals against avian influenza (3)
▢ Protection of myself or others against avian influenza (4)
▢ Animal welfare (5)
▢ Other, namely: (6) ________________________________________________

Q49 What would be reasons for you not to vaccinate your poultry against avian influenza?

*You may select multiple answers for this question.*

▢ No reasons against vaccination (1)
▢ I do not expect my poultry to become infected with avian influenza (2)
▢ I do not vaccinate my poultry because of my religion (3)
▢ I do not want to pay for a vaccine (4)
▢ I am concerned about side effects of the vaccine (5)
▢ I am concerned about strict monitoring requirements (6)
▢ Other, namely: (7) ________________________________________________

Q50 Would you be willing to have your poultry vaccinated against avian influenza?
If this becomes possible in the future.

|  | Not at all willing | Not willing | Neutral | Willing | Very willing |
| --- | --- | --- | --- | --- | --- |
| Willingness to vaccinate | ○ | ○ | ○ | ○ | ○ |

### 6.3 Information about previous bird flu infections

Q51 Has avian influenza been detected in your poultry?

*You do not have to answer this question if you prefer not to.*

o This has not been tested (1)
o No (2)
o Yes, in the past year (3)
o Yes, in the past five years (4)
o Yes, more than five years ago (5)
o Prefer not to say (6)

### 6.4 Information about the seasonal flu vaccination status of the poultry owner

Q52 Have you been vaccinated against influenza in the past year?

*You do not have to answer this question if you prefer not to.*

o Yes (1)
o No (2)
o Prefer not to say (3)

### **Final questions** Research of the Royal GD Animal Health

Q53 Are you participating in the Royal GD Animal Health study on animal diseases in hobby poultry?

*This is a different study from this questionnaire. The participants have already been selected. We are now asking whether you are one of them.*

o Yes (1)
o No (2)

*Display this question:*

*If Are you participating in the Royal GD Animal Health study on animal diseases in hobby poultry? = Yes (1)*

Q54 May we link your answers to this questionnaire to the test results from Royal GD Animal Health?

*We will link the test results to this questionnaire only for anonymised research. We will not share the test results with others.*

o Yes (1)
o No (2)

*Display this question:*

*If May we link your answers to this questionnaire to the test results from Royal GD Animal Health? = Yes (1)*

Q55 What is your postal code and house number?

*We use your postal code and house number only to link the results. We will not use this information for any other purposes. You can enter your postal code and house number in the following format:* *1234AB, 1.*

_____________________________

### Feedback and follow-up research

Q56 Do you have any questions, tips, or comments about this questionnaire?

_____________________________

Q57 May we contact you in the future for follow-up research?
If so, please enter your email address here.

_____________________________

### **End of Questionnaire**
