# Supplementary material for: Mapping the landscape of small-scale and backyard poultry flocks in the Netherlands: a nationwide survey for One Health surveillance
Source: One Health. 2026 Oct 23;23:101626. doi: 10.1016/j.onehlt.2026.101626 (PMC13763572; doi:10.1016/j.onehlt.2026.101626)
Supplement: Supplementary file 3 [file mmc3.docx]

### Manuscript: “Mapping the landscape of small-scale and backyard poultry flocks in the Netherlands: a nationwide survey for One Health surveillance”

Supplementary File B: Materials and methods

# Participant recruitment channels

The online questionnaire was disseminated using different channels, including the website of the Faculty of Veterinary Medicine of Utrecht University, the websites of various associations of backyard and small-scale animal keepers (e.g., *Small Pet Animal Enthusiasts Netherlands* (*KLN*)), and national veterinary organisations (e.g., *Group-practising veterinarians* (*CPD*) and the *Royal Netherlands Veterinary Association* (*KNMvD*)). Additionally, an announcement about the survey, including a QR-code directing participants to the online questionnaire, was published in newsletters of organisations such as the ***Association of City and Children’s Farms Netherlands* (*vSKBN*)** and the ***Federation of Care Farming* (*FLZ*)**. Furthermore, the survey was advertised on social media platforms, including LinkedIn, Instagram, and various Dutch small-scale poultry owner groups on Facebook. To reach a broader population of small-scale and backyard flock owners, the questionnaire was shared with the customer network of a retail chain specialised in gardening- and animal care products with locations throughout the Netherlands. Petting zoos and small-scale poultry farmers were contacted directly via their publicly available email addresses.

# Variable descriptions

### Holding types

In this study, we distinguished four different **holding types**: backyard flocks (backyard), petting zoos and care farms (petting & care farm), ornamental fowl breeders (breeder), and small-scale community farms (small community farm). Survey respondents were asked to self-identify their holding type, choosing one of the options listed above. Therefore, we refer to holding types as self-identified holding types. Participants were categorised into holding types, as we expected study outcomes to differ across holding types.

We defined backyard flocks as poultry kept as pet animals in private backyards. Flocks at petting and care farms are typically kept at locations with a large number of visitors. In addition, we expected multiple other (non-avian) animal species to be present at these sites. Flocks kept by breeders form a separate holding type, as we expected their motivations for poultry keeping, as well as their level of organisation, to differ from other holding types. Lastly, small community farmers were considered a distinct group based on their commercial activity and the larger flock sizes associated with this holding type.

### Poultry owner characteristics

Variables describing poultry owner characteristics were used to describe our target group. The characteristics included in the poultry owner description were **age**, **gender**, **education level**, whether poultry was kept at their property close to their home or elsewhere (**poultry at home**), whether respondents were affiliated with an animal organisation (**affiliated with association**), the number of other animal species they kept apart from birds (**number of other animals**), their geographical location (**postal code**), and the **urbanisation level** of the postal code region in which they kept their flock (which, in some cases, differs from where respondents live). Urbanisation levels (rural, suburban, and urban) were based on publicly available Statistics Netherlands (CBS) data (PC4 data) [1, 2]. We combined the levels with surrounding address densities of fewer than 500 addresses per km^2^ into “rural”, 500 to 1500 addresses per km^2^ into “suburban”, and 1500 addresses or above per km^2^ into “urban”. Address densities and corresponding urbanisation levels were defined for each postal code region of the Netherlands.

Poultry owner characteristics were compared to the general population (Table 1). Data of the general population were derived from CBS and the PC4 data [1, 3-5]. For education level, CBS-levels were reorganised into the levels corresponding to the poultry owner characteristics. For age, the <20 category was removed from the general population data to better align with the age distribution of the poultry owner data, as respondents were required to be at least 18 years old to participate in the study. The percentages for each remaining age category were normalised after excluding the <20 category. Additionally, we asked survey respondents to fill out an estimated number of neighbouring small-scale and backyard flocks within a 3 km radius from their own flock (**neighbouring flocks**), to obtain a better understanding of their spatial distribution.

Poultry owners’ **motivations** for poultry keeping are critical for understanding the perspectives of poultry owners, which are essential when evaluating the efficacy of, e.g., possible policy interventions. Additionally, as described in previous research, when investigating animal- and public health risks, understanding owners’ perspectives is helpful for managing those risks [6]. The variable **motivations** consisted of different categories: as a hobby or companionship, for egg production, nostalgic reasons, educational purposes, community feeling, breeding and attending exhibitions, poultry meat production, extra income generation, and ‘other’. The answers given by respondents were not mutually exclusive; thus, one poultry owner could report multiple motivations for poultry keeping.

### Flock size

**Flock sizes** were defined as the **total flock sizes**, including all captive birds (Galliformes, Anseriformes, raptors, and Ratitae) kept by the survey respondent. Flock sizes were categorised into four size categories: 1-10, 11-49, 50-249, and 250 birds and above. Flock size categories were based on findings from other studies, most of which reported median flock sizes smaller than 10 birds [7-10], or showed that at least the vast majority of holdings kept fewer than 50 birds [8, 11, 12]. Flock sizes were stratified by **holding type** as well as **urbanisation level**.

### Taxonomic bird groups

The captive bird species were divided into four different taxonomic **bird groups**: chickens, other non-chicken Galliformes (e.g., turkeys, guinea fowl, pheasants, or peacocks), Anseriformes (e.g., ducks, geese, and swans), and “other”. The other category included ratites, birds of prey, and, e.g., avocets and rail species. Most Galliformes species, especially chickens, are highly susceptible to AIV infections [13]. The presence of Anseriformes may increase the likelihood of AIV introduction from wild birds, while the co-housing of captive Anseriformes and Galliformes may facilitate within-holding transmission between these species. Since susceptibility to zoonotic pathogens such as AIV is dependent on bird species [13], it is important to understand which species are most commonly kept in small-scale and backyard holdings and whether keeping mixed flocks, including bird species from different bird groups, is a common practice. The co-housing of different bird groups was described by the variable **mixed housing**.

### Housing

Poultry **housing** is important for both poultry welfare and potential introduction risks of emerging pathogens, as it can influence direct and indirect exposure to wild birds, including environmental and fomite-mediated transmission [14-17]. In this study, we divided poultry housing into **indoor and outdoor housing**. Indoor housing was categorised into the following levels: a closed night shelter, an (half)-open night shelter, a mobile chicken car, no indoor (night) shelter, or other. Outdoor housing was categorised into the levels: free-range, a covered chicken coop, water (e.g., for waterfowl), no outdoor housing, or other.

# References

[1]	Statistics Netherlands (CBS), Four-digit postal code areas (numeric component) [dataset], Publieke Dienstverlening Op de Kaart (PDOK), Amersfoort, The Netherlands, 2023. https://service.pdok.nl/cbs/postcode4/atom/postcode4_numeriek_deel.xml.
[2]	Statistics Netherlands (CBS), Overview of all postal code areas with corresponding urbanisation category and address density (PC4stedoad): Documentation, Statistics Netherlands (CBS), The Hague/Heerlen, The Netherlands, 2024. https://geodata.cbs.nl/files/Postcode_stedelijkheid_OAD/Documentatie_PC4OADSTED.pdf.
[3]	Statistics Netherlands, Population; highest educational attainment and region [dataset], CBS StatLine, 2025. https://www.cbs.nl/nl-nl/cijfers/detail/85525NED.
[4]	Statistics Netherlands, Population; sex, age and marital status, 1 January [dataset], CBS StatLine, 2025. https://opendata.cbs.nl/#/CBS/nl/dataset/7461BEV/table.
[5]	Statistics Netherlands, Population Dashboard - Distribution, https://www.cbs.nl/nl-nl/visualisaties/dashboard-bevolking/verdeling/ (accessed 16 October 2026).
[6]	E.A. Buddle, K. Stevens, H.J. Bray, R.A. Ankeny, The chicken for the egg: Australian motivations for raising backyard chickens, Anthrozoös 38 (2) (2025) 299–310. https://doi.org/10.1080/08927936.2024.2449805.

[7]	M. Souvestre, M. Delpont, C. Guinat, C. Dumat, L. Guichard, L. Manis, et al., Backyard poultry flocks in France: A diversity of owners and biosecurity practices, Prev. Vet. Med. 197 (2021) 105511. https://doi.org/10.1016/j.prevetmed.2021.105511.
[8]	E. McClaughlin, S. Elliott, S. Jewitt, M. Smallman-Raynor, S. Dunham, T. Parnell, et al., UK flockdown: A survey of small-scale poultry keepers and their understanding of governmental guidance on highly pathogenic avian influenza (HPAI), Prev. Vet. Med. 224 (2024) 106117. https://doi.org/10.1016/j.prevetmed.2024.106117.

[9]	C. Correia-Gomes, N. Sparks, Exploring the attitudes of backyard poultry keepers to health and biosecurity, Prev. Vet. Med. 174 (2020) 104812. https://doi.org/10.1016/j.prevetmed.2019.104812.
[10]	A. Beam, L. Garber, J. Sakugawa, C. Kopral, Salmonella awareness and related management practices among small-scale or backyard poultry keepers, Prev. Vet. Med. 110 (3–4) (2013) 481–488. https://doi.org/10.1016/j.prevetmed.2013.02.007.

[11]	L. Pohjola, N. Tammiranta, C. Ek-Kommonen, T. Soveri, M.L. Hänninen, M. Fredriksson-Ahomaa, et al., A survey for selected avian viral pathogens in backyard chicken farms in Finland, Avian Pathol. 46 (2) (2017) 166–172. https://doi.org/10.1080/03079457.2016.1232804.

[12]	T. Zheng, B. Adlam, T.G. Rawdon, W.L. Stanislawek, S.C. Cork, V. Hope, et al., A cross-sectional survey of influenza A infection and management practices in small rural backyard poultry flocks in New Zealand, N. Z. Vet. J. 58 (2) (2010) 74–80. https://doi.org/10.1080/00480169.2010.65086.
[13]	K. Bertran, R. Dolz, N. Majo, Pathobiology of avian influenza virus infection in minor gallinaceous species: a review, Avian Pathol. 43 (1) (2014) 9–25. https://doi.org/10.1080/03079457.2013.876529.

[14]	J.L. Gonzales, S. Pritz-Verschuren, R. Bouwstra, J. Wiegel, A.R.W. Elbers, N. Beerens, Seasonal risk of low pathogenic avian influenza virus introductions into free-range layer farms in the Netherlands, Transbound. Emerg. Dis. 68 (1) (2021) 127–136. https://doi.org/10.1111/tbed.13649.
[15]	A. Hill, S. Gillings, A. Berriman, A. Brouwer, A.C. Breed, L. Snow, et al., Quantifying the spatial risk of Avian Influenza introduction into British poultry by wild birds, Sci. Rep. 9 (1) (2019) 19973. https://doi.org/10.1038/s41598-019-56165-9.
[16]	Y. Liu, L.J. Kjær, A.E. Boklund, C.K. Hjulsager, L.E. Larsen, C.T. Kirkeby, Risk factors for avian influenza in Danish poultry and wild birds during the epidemic from June 2020 to May 2021, Front. Vet. Sci. 11 (2024) 1358995. https://doi.org/10.3389/fvets.2024.1358995.

[17]	F.C. Velkers, T.T.M. Manders, J.C.M. Vernooij, J. Stahl, R. Slaterus, J.A. Stegeman, Association of wild bird densities around poultry farms with the risk of highly pathogenic avian influenza virus subtype H5N8 outbreaks in the Netherlands, 2016, Transbound. Emerg. Dis. 68 (1) (2021) 76–87. https://doi.org/10.1111/tbed.13595.
