# Supplementary material for: Mapping the landscape of small-scale and backyard poultry flocks in the Netherlands: a nationwide survey for One Health surveillance
Source: One Health. 2026 Oct 23;23:101626. doi: 10.1016/j.onehlt.2026.101626 (PMC13763572; doi:10.1016/j.onehlt.2026.101626)
Supplement: Supplementary file 4 [file mmc4.docx]

### Manuscript: “Mapping the landscape of small-scale and backyard poultry flocks in the Netherlands: a nationwide survey for One Health surveillance”

Supplementary File C: Supplementary figures

**Fig. C1:** A) Model-based survey respondent intensity, presented as relative risks (log-scale) per postal code region based on expected respondents by postal code surface size in km^2^. Postal code regions in red represent areas with an on average large number of respondents per km^2^. Multiple neighbouring, red-coloured postal codes visualise potential clustering of respondents. Postal code regions in dark teal represent areas with an overage low number of respondents per km^2^. B) Self-reported estimations of the neighbouring backyard flock density (within a 3 km radius) (log-scale). Each postal code region represents the average of the estimations provided by all survey respondents residing in the corresponding postal code region. Regions in red represent postal codes with a relatively high density of neighbouring holdings. Postal code regions coloured dark teal represent areas with a relatively low (to 0) density of neighbouring holdings. Regions for which data were missing are not coloured (white). C) Respondent discordance: postal codes with a low model-based survey respondent intensity (low relative risk) but with a relatively high self-reported average estimated number of neighbouring backyard flocks in the surroundings. Postal codes in dark teal represent areas with potential undersampling of small-scale and backyard poultry owners. Postal codes in beige represent regions included in the correlation-analysis that do not show discordance. Regions for which data were missing are not coloured (white).
